# Ascorbic Acid, Ultraviolet C Rays, and Glucose but not Hyperthermia Are Elicitors of Human *β*-Defensin 1 mRNA in Normal Keratinocytes

**DOI:** 10.1155/2015/714580

**Published:** 2015-03-01

**Authors:** Luis Antonio Cruz Díaz, María Guadalupe Flores Miramontes, Paulina Chávez Hurtado, Kirk Allen, Marisela Gonzalez Ávila, Ernesto Prado Montes de Oca

**Affiliations:** ^1^Molecular Biology Laboratory, Biosecurity Area, Pharmaceutical and Medical Biotechnology Unit (UBMF), Research Center in Technology and Design Assistance of Jalisco State (CIATEJ, AC), National Council of Science and Technology (CONACYT), Guadalajara, JAL, Mexico; ^2^In silico Laboratory, UBMF, CIATEJ AC, CONACYT, Guadalajara, JAL, Mexico; ^3^Lancaster Medical School, Lancaster University, Lancaster, UK; ^4^Human Digestive Tract Simulator, UBMF, CIATEJ, AC, CONACYT, Guadalajara, JAL, Mexico

## Abstract

Hosts' innate defense systems are upregulated by antimicrobial peptide elicitors (APEs). Our aim was to investigate the effects of hyperthermia, ultraviolet A rays (UVA), and ultraviolet C rays (UVC) as well as glucose and ascorbic acid (AA) on the regulation of human *β*-defensin 1 (*DEFB1*), cathelicidin (*CAMP*), and interferon-*γ* (*IFNG*) genes in normal human keratinocytes (NHK). The indirect *in vitro* antimicrobial activity against *Staphylococcus aureus* and *Listeria monocytogenes* of these potential APEs was tested. We found that AA is a more potent APE for *DEFB1* than glucose in NHK. Glucose but not AA is an APE for *CAMP*. Mild hypo- (35°C) and hyperthermia (39°C) are not APEs in NHK. AA-dependent *DEFB1* upregulation below 20 mM predicts *in vitro* antimicrobial activity as well as glucose- and AA-dependent *CAMP* and *IFNG* upregulation. UVC upregulates *CAMP* and *DEFB1* genes but UVA only upregulates the *DEFB1* gene. UVC is a previously unrecognized APE in human cells. Our results suggest that glucose upregulates *CAMP* in an IFN-*γ*-independent manner. AA is an elicitor of innate immunity that will challenge the current concept of late activation of adaptive immunity of this vitamin. These results could be useful in designing new potential drugs and devices to combat skin infections.

## 1. Introduction

Keratinocytes control skin microbial colonization/infection in part by synthesizing human *β*-defensin 1 (hBD-1) and cathelicidin LL-37, both of which are wide-spectrum antimicrobial peptides [1–4]. Antimicrobial peptide elicitors (APEs) are defined as physical (class I), chemical (class II), and biological (class III) agents that promote upregulation of endogenous antimicrobial peptides (APs) [5–8].

*IFNG* is a relevant gene in innate and adaptive responses; specifically, its product, IFN-*γ*, is an APE for both* CAMP* (codes for LL-37) and* DEFB1 *(codes for hBD-1) in monocytes and gingival keratinocytes, respectively [6]. In this report we wanted to know if keratinocytes upregulate* IFNG* in response to APE, independently of the* IFNG* mRNA provided by lymphocytes in adaptive immunity. Probable APEs with potential application in skin therapy against infections in humans are hyperthermia, ultraviolet A rays (UVA), ultraviolet C rays (UVC), ascorbic acid (AA or vitamin C), and glucose.

In a mice model of influenza infection, hyperthermia, a potential class I APE, is beneficial because it increases leukocyte count and diminishes proinflammatory cytokines, presumably avoiding damage to infected tissue [9]. In humans, hyperthermia is beneficial in treating several diseases such as neurosyphilis, some forms of chronic arthritis, and cancer. Furthermore, many infections cause fever during certain phases, including fever caused by* Rickettsia *sp.,* Chlamydia *sp., viruses, or parasites. When this fever is associated with endogen pyrogen, it leads to the activation of T cells which enhances the host's defense system [10].

UVA (320–400 nm) are APEs of* DEFB1* in scleroderma skin lesions [11] but they have not been tested in normal keratinocytes. UVC (180–280 nm) have been suggested as a prophylactic approach in a mice model of infection against* Pseudomonas aeruginosa* and* Staphylococcus aureus* [12] but the function as an APE of this wavelength has not been described.* S. aureus* is a very common human pathogen susceptible to LL-37 and hBD-1 [6]. In the case of* Listeria *sp., the pathogen is susceptible to LL-37. These APEs have a therapeutic potential against this pathogen in epithelial cells [13, 14]. In this study, we indirectly assume that microbicidal activity is due the increment of the level of synthesis of these peptides by the APEs as suggested by the mRNA levels and inhibition zones of the antimicrobial assays.

AA has been widely used to prevent and treat the common cold, malaria, and diarrhea infections and pneumonia. AA improves the efficacy of antimicrobial and natural killer cell (NK) activities [15]. Whether this antimicrobial activity is dependent on induction and/or upregulation of APs is unknown. Glucose is an APE of* DEFB1* in kidney and colon cells [16] but its effect on both* DEFB1 *and* CAMP* in keratinocytes is unknown.

In this study we assessed the capacity of UVA, UVC, AA, glucose, and hyperthermia to act as APEs of* DEFB1* and* CAMP* genes and as inductors of* IFNG* in a cell line of neonate normal human keratinocytes (NHK).

## 2. Materials and Methods

### 2.1. Cell Culture and Exposure to Potential APEs

Normal human neonate keratinocytes (ATCC PCS-200-010) were maintained in K-SFM cell culture media (Gibco) at 37°C in a humidified incubator with 5% CO_2_. We exposed 1 × 10^6^ cells to D-(+)-glucose (5.5, 15.5, 25.5, and 45.5 mM, Sigma, number of catalog: G8644) or L-ascorbic acid (0, 5, 10, and 20 mM, Sigma, number catalog: A4544) for 24, 48, and 72 h. The incubation temperature was also assessed as potential APE mimicking human mild hypothermia (35°C), normothermia (37°C), and hyperthermia (39°C)* in vitro.* Additionally, for the ascorbic acid at 20 mM, the interaction between time (24, 48, and 72 h) and temperature (35, 37, and 39°C) was assessed. The exposition to UV rays (UVA at 365 nm, UVC at 254 nm) was performed with a square source of irradiation of 4 Watts (UV lamp UVP-UVGL-25, UVP, Cambridge, UK, number of catalog: 95-0021-12) at 9.2 cm from the adhered cells.

### 2.2. Quantitative Real-Time PCR (qPCR)

Nucleic acids were extracted in a MagNA Pure LC 2.0 Instrument (Roche) with the MagNA Pure LC total nucleic acid kit (Roche, catalog number 03038505001). Purified RNA was DNAse-treated and reverse transcribed with a mix containing random primers (Invitrogen) and ArrayScript enzyme (Applied Biosystems). Quantitative real-time PCR was performed with 33 ng cDNA with Fast SYBR Green Master Mix (Applied Biosystems). The reactions were run in a Step One Thermocycler (Applied Biosystems) using the following primers sequences designed with Primer Express v. 3.0 software (Applied Biosystems) and tested for specificity in PRIMER-BLAST (http://www.ncbi.nlm.nih.gov/tools/primer-blast/): for* HPRT *(endogenous control suggested for normal human keratinocytes according to Allen et al. [17]), F 5′-TGTTCAAATTATTACCAGTGAATCTTTGTC-3′, R 5′ TTTTAAATTTTTGGGAATTTATTGATTTG-3′; for* IFNG* F 5′-GCTGACTAATTATTCGGTAACTGACTTG-3′, R 5′-TAGCTGCTGGCGACAGTTCA-3′; for* DEFB1* F 5′-GAGAACTTCCTACCTTCTGCTGTTTAC-3′, R 5′-AAAGTTACCACCTGAGGCCATCT-3′; for* CAMP* F 5′-ACCCAGACACGCCAAAGC-3′, R 5′-TTCACCAGCCCGTCCTTCT-3′. The PCR program was 95°C for 20 s, 40 cycles of denaturing at 95°C for 3 s, and annealing/extension at 60°C for 30 s. Melting curves were performed at 95°C for 15 s, 60°C for 1 min, and 95°C for 15 s. Amplification efficiencies for each gene were calculated and the relative quantitative expression was obtained according to the Pfaffl method [18]. The normalization of gene expression included the input RNA and input cell count. The controls were samples with the vehicle at 24 h, or in the case of temperatures the normothermic sample (37°C) for each day.

### 2.3. Antimicrobial Assays

Diffusion assays were performed according to Kirby et al. [19]. We tested culture supernatants samples with a volume of 50 *μ*L poured into 6 mm diameter wells. The negative control was the supernatant of the culture medium without the potential APE and the positive control was 2.25 *μ*M (1 mg/mL) of tetracycline. Three biological replicas of each were performed with strains of* Listeria monocytogenes *(ATCC 19114, susceptible to LL-37 [13, 14]) and* Staphylococcus aureus* (ATCC 25923, susceptible to LL-37 and hBD-1) [6].

### 2.4. Cytotoxicity Assays

In the experimental conditions where gene upregulation was observed, experiments were repeated and cytotoxicity assays were performed in increasing concentrations of the APEs. This was assessed by applying 0.05% trypsin by 12 min and neutralizing with DMEM (10% fetal bovine serum). After centrifugation (400 ×g, 5 min), three 10 *μ*L aliquots of homogeneous precipitated cells were quantified in a Neubauer chamber. The percentage of viable cells adhered to the plate was compared with the control without elicitor, considered as being 100% viable. Quantifications of each day were compared with the control of the corresponding days (Cruz Díaz et al., in process).

### 2.5. Statistical Analysis

Statistical comparisons were done in Microsoft Excel 2010. To evaluate if variances were different or equal among treatments, we performed *F* tests. Depending of the results of *F* tests, Student's *t*-tests for equal or nonequal variances were performed. Results are shown as mean + standard error of the mean (SEM). All experiments were performed at least in triplicate, and a *P* value <0.05 was considered statistically significant.

## 3. Results

**Figure 1:**
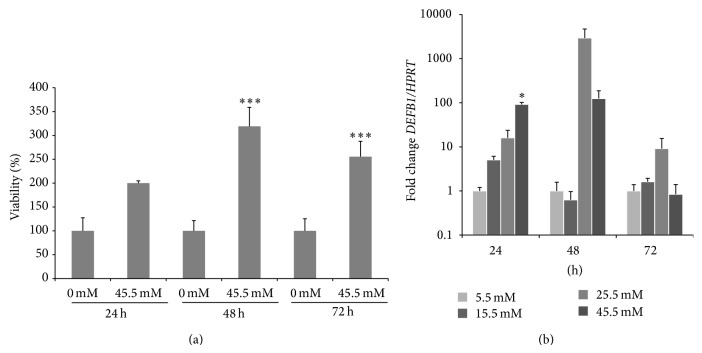
Effect of glucose in normal human keratinocytes. (a) Viability percentage of keratinocytes to glucose exposition by 24 h, 48 h, and 72 h in comparison with their controls for each exposure time. (b)* DEFB1* expression at 24, 48, and 72 h of keratinocytes exposed to glucose. Reference group was 5.5 mM (normal concentration of culture medium) at each time. Error bars represent SEM and significant values of Student's *t* tests are depicted as ^*^ *P* < 0.05; ^***^ *P* < 0.001.

When we exposed the dermal cells to increasing levels of glucose, simulating increased absorption and distribution of glucose after feeding, the viability of keratinocytes increased at 24 h and was found to be highly significant at 48 h and 72 h (Figure 1(a)). At 24 h of glucose exposure (45.5 mM),* DEFB1 *expression was significantly upregulated (Figure 1(b)).

**Figure 2:**
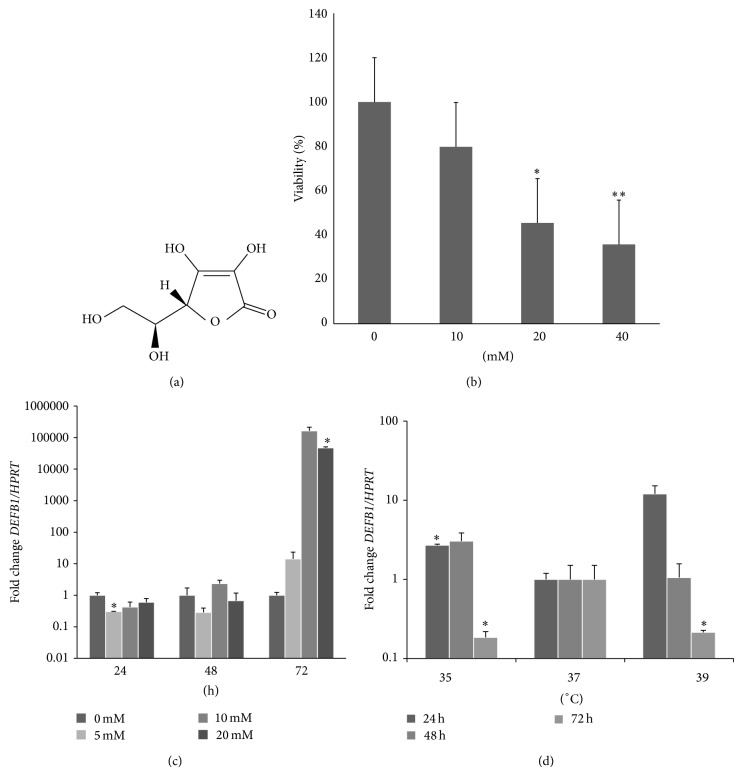
Effect of ascorbic acid alone or in combination with hypothermia or hyperthermia in normal human keratinocytes. (a) Structure of L-ascorbic acid. (b) Cytotoxicity assays exposed 24 h with ascorbic acid. (c) Effect of different concentrations of ascorbic acid on* DEFB1* expression at 24, 48, and 72 h. The reference group was 0 mM at each time. (d) Effect of hypothermia, normothermia, and hyperthermia, all plus interaction with 20 mM ascorbic acid on* DEFB1* expression. Reference group was normothermia (37°C) at each time. Error bars represent SEM and significant values of Student's *t* tests are depicted as ^*^ *P* < 0.05; ^**^ *P* < 0.01.

The viability of keratinocytes is not affected at 10 mM of ascorbic acid (AA, Figure 2(a)) but begins to be affected at 20 mM and was highly significantly affected at 40 mM (Figure 2(b)). When keratinocytes were exposed to 10 and 20 mM of ascorbic acid,* DEFB1* was upregulated by almost 5 logs after 72 h (Figure 2(c)).

**Figure 3:**
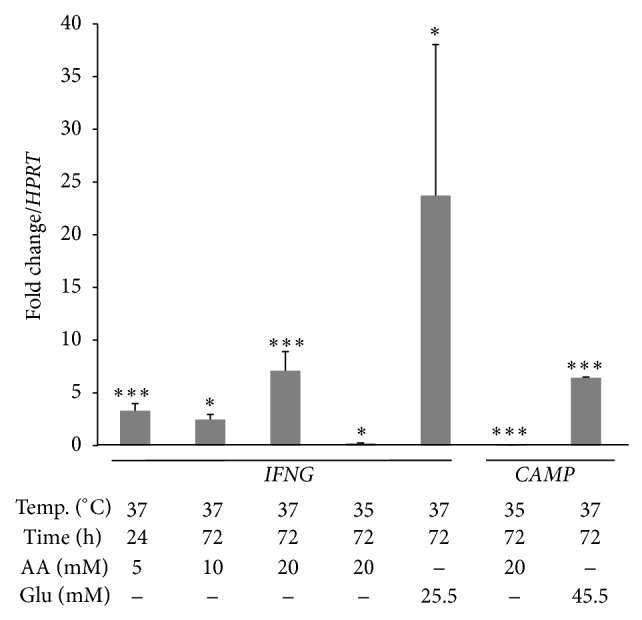
Statistically significant results of the effect of ascorbic acid, hypothermia, and/or glucose exposure in* IFNG *and* CAMP *expression in normal human keratinocytes. Error bars represent SEM and significant values of Student's *t* tests are depicted as ^*^ *P* < 0.05; ^***^ *P* < 0.001.

At 72 h rather than at 24 h, glucose also upregulated* IFNG *and* CAMP *genes (Figure 3). AA also upregulated* IFNG* (Figure 3), but AA did not affect* CAMP* expression at any time or concentration (data not shown).* DEFB1 *(3 logs),* CAMP* (1 log), and* IFNG* (1 log) were upregulated in both hypothermia and hyperthermia conditions mainly at 72 h, although the results were not significant (data not shown). We then assessed the effect of temperature at 20 mM of AA by 72 h (highest significant upregulation of* DEFB1*, Figure 2(c)). Compared with normothermia,* DEFB1* was upregulated at 24 h at 35°C but downregulated at 72 h at both 35 and 39°C (Figure 2(d)). Interestingly, at hypothermia and 20 mM of AA both* IFNG *and* CAMP *were downregulated at 72 h (Figure 3). Furthermore, the expressions of* DEFB1* and* IFNG *correlate linearly with 20 mM at 35°C (*r* ^2^ = 0.9).

**Figure 4:**
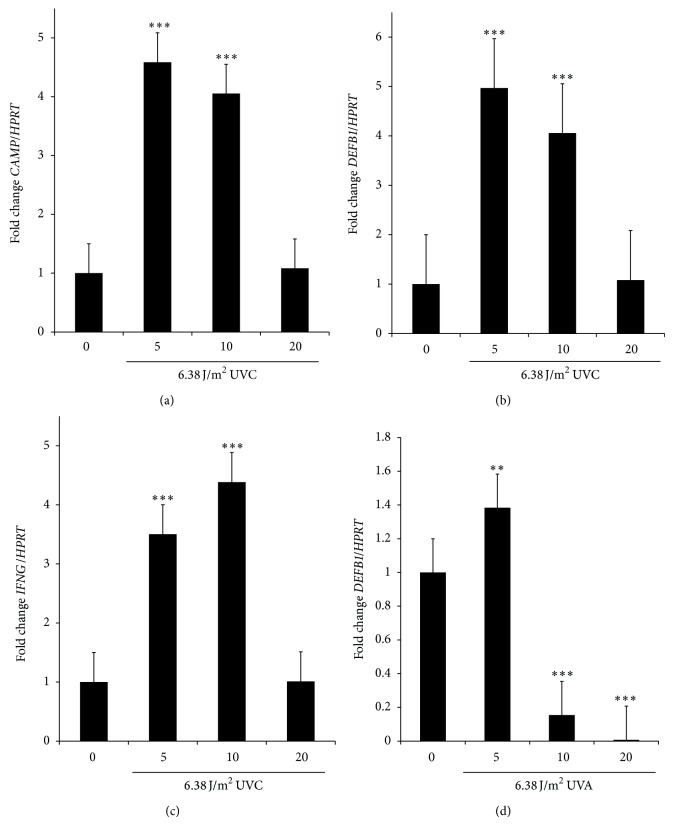
Relative gene expression in keratinocytes exposed to UVC (254 nm) and UVA (365 nm). (a)* CAMP *expression in UVC exposure. (b)* DEFB1* expression in UVC exposure. (c)* IFNG *expression in UVC exposure. (d)* DEFB1* expression in UVA exposure. Exposure is expressed in minutes. In all cases reference group was 0 min (not exposed). Error bars represent SEM and significant values of Student's *t* tests are depicted as ^**^ *P* < 0.01; ^***^ *P* < 0.001.

UVC upregulated* CAMP* at 5 and 10 min of exposure (Figure 4(a)) as well as upregulating* DEFB1* (Figure 4(b)) and* IFNG* genes (Figure 4(c)). Surprisingly, UVA slightly upregulated* DEFB1* at 5 min of exposure and downregulated it at 10 and 20 min of exposure (Figure 4(d)). UVA do not impact* CAMP* or* IFNG* regulation (data not shown).

**Figure 5:**
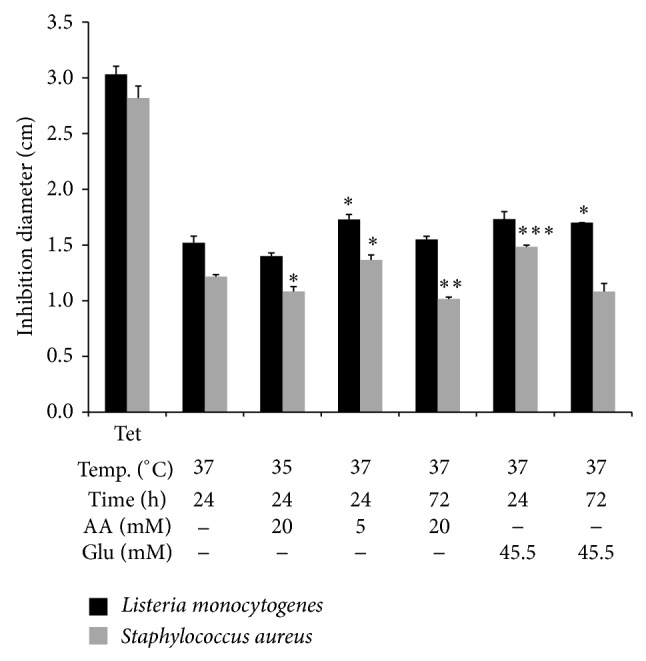
Antimicrobial diffusion assays with the supernatants of APEs-exposed keratinocytes. Tet, tetracycline, was included as a positive control (2.25 *μ*M, 1 mg/mL). The negative control was supernatants of cells without elicitor (37°C, 24 h). Error bars represent SEM and significant values of Student's *t* tests are depicted as ^*^ *P* < 0.05; ^**^ *P* < 0.01; ^***^ *P* < 0.001.

The supernatant of NHK in the condition 5 mM AA, 24 h, 37°C, that shows upregulation of both* CAMP* (2.56-fold) and* IFNG *(3.31-fold) showed an increased antimicrobial activity against both* L. monocytogenes *and* S. aureus *(Figure 5) in spite the diminished expression of* DEFB1 *(0.5-fold). As expected due to diminished viability, both conditions 37°C, 20 mM AA, 72 h, and 35°C, 20 mM AA, 24 h, diminish the activity against* S. aureus* significantly even at the former condition which had the highest* DEFB1* expression (4 logs).

Both samples from 45.5 mM glucose, 24 h and 72 h, showed increased antimicrobial activity against* S. aureus *and* L. monocytogenes *respectively, as expected, presumably because in these samples* DEFB1* (90-fold) and* CAMP *(6.43-fold) genes were upregulated, respectively.

## 4. Discussion

We demonstrate for the first time that AA is an APE for* DEFB1* but not for* CAMP*. We therefore propose that the indirect antimicrobial effect of ascorbic acid in NHK could be at least partially dependent on* DEFB1* induction which challenges the traditional view regarding AA only as being considered a player in the later activation of the adaptive immunity [20]. This also could explain why AA acts as an antitumor agent [21–23], since* DEFB1* is considered a tumor suppressor gene [24, 25].

Regarding cytotoxicity assay, Trypan Blue Exclusion (TBE) Assay overestimates viable cells [26] and assignment of trypan-stained cells to viable or nonviable categories was found to be subjective and arbitrary [27]. Regarding 3-(4,5-dimethylthiazol-2-yl)-2,5-diphenyltetrazolium bromide (MTT) assay, a lineal relationship exists between reduced tetrazolium dye and cell number only up to 2 × 10^4^ cells/well with no relationship at higher cell numbers and/or absorbance values of greater than 0.8 [27]. Also MTT was designed to test cytotoxic drugs and the recommended set of concentrations must include the highest concentration that kills most of the cells and the lowest concentration that kills none of the cells [28]. The main advantages of the proposed protocol is that in adherent cultures it permits to obtain a number of viable cells that are already attached, because the nonattached cells, if any, are removed by aspirating the supernatant. With our method, there is less ambiguity assigning viability as in TBE and chemical reactions even in the absence of viable cells. This does not affect the results as in MTT (Cruz Díaz et al., in process).

It has been reported that short-term hypothermia (taken as 30–34°C, 2–4 hours,) in cell lines and murine models increases the level of anti-inflammatory cytokines (IL-4 and IL-10) and decreases proinflammatory cytokines (IL-1, IL-2, IL-6, and TNF-*α*), inhibiting lymphocyte proliferation and decreasing HLA-DR expression associated with cell activation. Long term (>24 hours) hypothermia, however, increases proinflammatory cytokine levels [29]. The hyperthermic conditioning (41°C) has been an effective treatment in mice with sepsis in combination with LL-37. In rats with hypothermia (32°C) prior to sepsis, the IL-10 levels were significantly increased compared to normothermic rats, altering the cytokines profile, survival, and recruitment of granulocytes suggesting immunosuppression [30]. Human hypothermia (32°C) is associated with elevated frequency of infectious complications; dysfunction of the immune response caused by hypothermia has been demonstrated in both clinical and animal studies [31]. Contrary to our hypothesis, hyperthermia does not upregulate the expression of* DEFB1* or* CAMP* in a significant manner; nevertheless the strong tendency to upregulate mainly* DEFB1 *(data not shown) deserves further investigation.

On the contrary, we observed that hypothermia improved the potential of AA (20 mM) both acting as APE at 24 h. However, in this combination, both extreme conditions (hypo- and hyperthermia) diminished* DEFB1* expression at 72 h. This could be because in these two scenarios the keratinocytes are exposed to a prolonged oxidative stress probably requiring more AA to counteract the adverse environment. Notably, the upregulation of* DEFB1* with AA does not predict antimicrobial activity* in vitro *probably because in an amount of equal to or higher than 20 mM we found that AA is cytotoxic. Also, high oral doses of AA* in vivo* cause hyperoxaluria, which is the excessive urinary excretion of oxalate and often the formation of kidney stones [10]. Clinical trials must uncover the optimal doses of AA in fever episodes of diverse infections to reach the expected efficacy of AA as APE of* DEFB1*. The role of AA as APE was anticipated because AA reduces the expression of IL-10 in a dose-dependent way [32] and this cytokine inhibits the expression of hBD-1 [33].

According to our results,* IFNG* mRNA levels probably provided the keratinocyte with an innate immunity against skin pathogens, independent of the late lymphocyte-derived IFN-*γ* (adaptive immunity) in an established infection [34]. Also we found that IFN-*γ* is upregulated earlier than* DEFB1 *suggesting that the former could act as an APE in NHK of foreskin as is true in gingival keratinocytes [35, 36], macrophages [37], and monocytes [38]. The condition where* IFNG* and* CAMP* were upregulated was the only one that predicts increment antimicrobial activity in the two tested Gram-positive pathogens, suggesting that this* IFNG*-dependent response is a stronger APE than* IFNG*-independent response (e.g., TNF-*α*-mediated) [6]. The antimicrobial* in vitro* prediction of* CAMP* upregulation is in concordance with a recent demonstration that transfecting* CAMP *mRNA increases resistance to* Listeria *sp. in oral epithelial cells [14].

In diabetic and obese rat models, the expression of *β*-defensin 1 (BD-1) was lower than that of slim rat controls [39] and only *β*-defensin 2 was found to be increased in diabetic rats, most likely as a result of a proinflammatory response [40]. Glucose has been described as an APE of* DEFB1* in human embryonic kidney HEK-293 [16, 41] and colon adenocarcinoma cells HCT-116 [16] but not for keratinocytes. Our results suggest an impact of intracellular glucose deficiency in susceptibility to skin infections due to* DEFB1* downregulation, for example, ulcers in diabetic patients. We also demonstrate that glucose induces a faster and higher expression of* DEFB1* compared to* CAMP* in normal keratinocytes. The glucose-dependent expression of* DEFB1* and* CAMP* has a biological relevance because they show constitutive skin expression, probably explained by the constant supply of glucose through blood in a nonstarved state and/or normal internalization of glucose in a nondiabetic host. Interestingly, Barnea et al. did not find dose-dependent response above 10 mM of glucose in HEK-293 cells [16] as we found in NHK, revealing a possible higher tissue-specific response. Furthermore, some authors consider that* CAMP* is an inducible gene. However in our system of NHK from neonate foreskin it always showed a constitutive expression as previously suggested in squamous epithelia of mouth, tongue, esophagus, vagina, and cervix [42].

UVA makes up 95% of UV light that reaches the earth's surface [43]. UVA inhibits cell proliferation due to the arrest of the S-phase [44] and also acts as an inhibitor of* DEFB1* in scleroderma lesional skin, with no effect in unaffected skin in these patients [11]. On the contrary, we found that in NHK UVA is a modest APE of* DEFB1 *but an antimicrobial peptide inhibitor (API) at longer exposures. These suggest that in scleroderma lesions the potential of this APE could be affected by an alteration of* DEFB1* overexpression pathways [7, 45] probably due to a unbalanced cytokine expression profile [6]. The lack of upregulation of* IFNG* in the exposure to UVA suggests that the route of* DEFB1* expression with this APE is IFN-*γ*-independent, but this deserves further investigation.

Our results reinforce and expand the notion that UV light (beyond UVA and UVB) stimulates APs gene expression. Furthermore, we were able to induce upregulation of* DEFB1* and* CAMP* genes at 4.25% of energy and 0.347% of exposure time required to upregulate* DEFB4, DEFB103*, and* S100A7* genes [46]. In spite of the fact that in nature most of UVC is usually blocked by the stratospheric ozone layer [43], compact fluorescent light bulbs expose our skin to UVC as well as UVA [44]. UVC causes degradation of I*κ*B*α* and nuclear entry of p65/RelA thus activating the NF-*κ*B pathway (independent of I*κ*B kinase-IKK-activation) [47]. This nuclear factor is a probable regulator of* DEFB1 *expression as suggested by our group [48]. We demonstrate for the first time that UVC in short exposures can be useful as APE of* CAMP* and* DEFB1*, even though it is well known that longer exposure to UVC causes DNA damage [44].

## 5. Conclusion

Mild hypo- (35°C) and hyperthermia (39°C) are not APEs in NHK* in vitro*, even when hyperthermia shows a tendency to act as an APE with probable biological significance, in spite of its not being statistically significant. Moderate exposure to UVC upregulates* CAMP* and* DEFB1 *genes, but UVA only upregulates the* DEFB1 *gene in HNK at 5 min of exposure. UVA is an API of* DEFB1 *at longer exposures. The later apparently downregulation could be explained because mRNA of* DEFB1* start degrading after 5 min of exposure to UVA. But this requires further investigation. These results suggest alternative expression pathways or different induction thresholds for* DEFB1* and* CAMP*. UVC is a previously unrecognized APE in human cells. These findings uncover the potential of novel therapeutics in skin infectious diseases.

Our results also suggest that glucose upregulates* CAMP* in an IFN-*γ*-independent manner and thus probably in an inflammation-independent manner. AA is a more potent APE for* DEFB1* than glucose in NHK. Glucose but not AA is an APE for* CAMP*. AA-dependent* DEFB1* upregulation below 20 mM predicts* in vitro* antimicrobial activity as well as glucose- and AA-dependent* CAMP* and* IFNG* upregulation. Levels of serum glucose in diabetic patients could be useful to predict susceptibility to infections due to diminished APs expression, but this requires further clinical studies. AA as APE will challenge the current concept of late activation of adaptive immunity of this vitamin probably acting at the keratinocyte level independent of adaptive immunity. This knowledge could be useful to develop new potential drugs and devices to combat skin infections based on these APEs. Future research in classes I and II APEs holds great promise.
